# Enhancement of nutritional and functional qualities of tropical leaf meal as feed ingredients in chickens through the use of fermentation technology

**DOI:** 10.1007/s11250-024-04223-4

**Published:** 2024-11-09

**Authors:** I. P. Ogbuewu, C. A. Mbajiorgu

**Affiliations:** 1 Department of Animal Science and Technology, Federal University of Technology, P.M.B. 1526, Owerri, Imo State Nigeria; 2 Department of Agriculture and Animal Health, University of South Africa, Florida Science Campus, Private Bag X6, Florida, 1710 South Africa

**Keywords:** Tropics, Leaf meals, Fermentation, Nutrient enrichment, Chicken performance

## Abstract

The poultry industry in developing countries is challenged by the high cost of conventional protein and energy feed ingredients. This problem has burdened researchers to use cheap and readily non-conventional feed ingredients such as tropical leaf meals (TLMs) to reduce the cost of feed. Tropical leaf meals are high in nutrients and important bioactive compounds, such as flavonoids and polyphenols. These important bioactive compounds in TLMs are responsible for their health promoting effects in animals. Research has shown that inclusion of moderate quantities of TLMs in livestock feed improves chicken performance and health. However, the inclusion of high levels of TLMs in poultry diets reduced chicken performance, which may be attributed to low palatability, high fibre content, poor digestibility, and the presence of antinational factors (ANFs) in TLMs. The potential of fermentation to enhance the nutrient content of feedstuffs high in fibre has been reported. Therefore, the objective of this research was to review the current knowledge on the effect of fermentation on nutritional and functional properties of TLMs and their feeding value on broiler chicken and laying hen performance.

## Introduction

The modern poultry industry in developing nations is constrained by the rising cost of conventional feedstuffs (Ahiwe et al. 2022). Thus, there is a need to find close alternatives to conventional feedstuffs using tropical leaf meals (TLMs). According to Okoli et al. (2014), TLMs are high in nutrients and nutraceuticals, thus, making them suitable for use as feed ingredients in poultry diets (Ogbuewu and Mbajiorgu 2019; Okoli et al. 2019; Ogbuewu et al. 2023). Importantly, TLMs have few food and industrial values, making them readily available as poultry feedstuffs. Examples of tropical plants whose leaves have received attention in poultry production include cassava (*Manihot spp.*), mango (*Magnifera indica*), Bushbuck (*Gongronema latifolium*), pawpaw (*Carica papaya*), neem (*Azadirachta indica*), Microdesmis (*Microdesmis puberula*), and several others (Ogbuewu and Mbajiorgu 2019, 2023a; Okoli et al. 2019). However, low palatability, high fibre content, poor digestibility, and the presence of ANFs in TLMs have limited their use in chicken diets (Achonwa et al. 2019; Ogbuewu and Mbajiorgu 2019). Evidence exists that ANFs cause pathological changes in the chicken gut (Yamamoto et al. 2010), thereby reducing nutrient absorption and uptake (Nordrum et al. 2000).

Techniques including drying, milling, and fermentation have been employed to improve nutrient content and functional properties of novel feedstuffs that are high in ANFs, fibre, and low in protein (Aladi et al. 2017; Chukwukaelo et al. 2018; Ogbuewu and Mbajiorgu 2023b). Fermentation has been reported to increase the nutrient content of feed materials through the synthesis of extracellular enzymes, production of the bioactive substances, and the bioavailability of vital nutrients (Bao et al. 2020; Wang et al. 2021). Fermentation has also been reported to stimulate the growth of lactic acid bacteria (LAB), reduce pH, and increase the concentration of organic acids in feed materials (Bao et al. 2020; Wang et al. 2021). These latter features have been found to protect finished feed from microbial spoilage and improve gut microbiota composition and nutrient utilisation in several livestock species (Melini et al. 2019; Sugiharto and Ranjitkar 2019; Mapelli-Brahm et al. 2020). The proliferation of LAB during fermentation encourages the conversion of total phenols into more active metabolites, which react with anthocyanidins to produce alkyl catechols that can protect the cells from oxidative damage (Mapelli-Brahm et al. 2020). In the context of cost efficiencies, the substitution of expensive conventional feedstuffs in poultry diets with less expensive novel feed resources such as TLMs could further promote the use of less expensive novel fermented feed resources in chicken nutrition. Based on the aforementioned qualities of TLMs, this review aims to highlight the potential of fermentation to enhance the nutrient content and functional qualities of TLMs, hence enhancing their utilisation in chicken nutrition.

## Fermentation

Fermentation is a chemical process in which microbes degrade complex substrates into simple substances that can be readily utilised by chickens (Chukwukaelo et al. 2018; Sugiharto and Ranjitkar 2019). Fermentation products differ depending on fermentation conditions (temperature, humidity), fermentation type (aerobic or anaerobic), microbes used, and substrate properties (Aladi et al. 2017; Sugiharto and Ranjitkar 2019). Fermentation techniques are classified into two types: solid-state fermentation (SSF) and submerged fermentation (SmF) (Sugiharto and Ranjitkar 2019). The SmF method involves the use of excess liquid substrates such as molasses, fruit juice, whey, nutrient broth, vegetable juice, and wet distiller grains (Sugiharto et al. 2015) and is the most common technique for producing probiotics in the industry. Often, submerged fermentation is used to generate fermented liquid feed, in which the finished feed is fermented after being mixed with water or liquid by-products from the brewing industry (Sugiharto and Ranjitkar 2019). Unlike the SmF technique, SSF entails the fermentation of solid substrates in the absence of free-flowing liquid (Aladi et al. 2017; Chukwukaelo et al. 2018). This technique is used to produce dry fermented feed that is incorporated into finished feed. Due to limited moisture levels, SSF can only be done by a limited number of microbes, mainly fungi (*Aspergillus spp*.) and some bacteria like Lactobacillus (Supriyati et al. 2015). Bhanja et al. (2016) found that SSF is better than SmF due to lower water and energy requirements, excellent aeration, resemblance to the natural habitat of microbes, and eco-friendliness.

To date, both SSF and SmF methods are used to produce poultry feeds depending on the substrate types (Missotten et al. 2013; Aladi et al. 2017; Sugiharto and Ranjitkar 2019). According to Shim et al. (2010), SSF is more commonly used in the production of fermented feeds than SmF, as this technique produces products with higher yield and quality. The high moisture level in fermented feeds made from SmF may pose a problem for chicken production, in the context of practical feeding and litter management (Engberg et al. 2009).

### Characteristics of fermented traditional feed resources

**Fig. 1:**
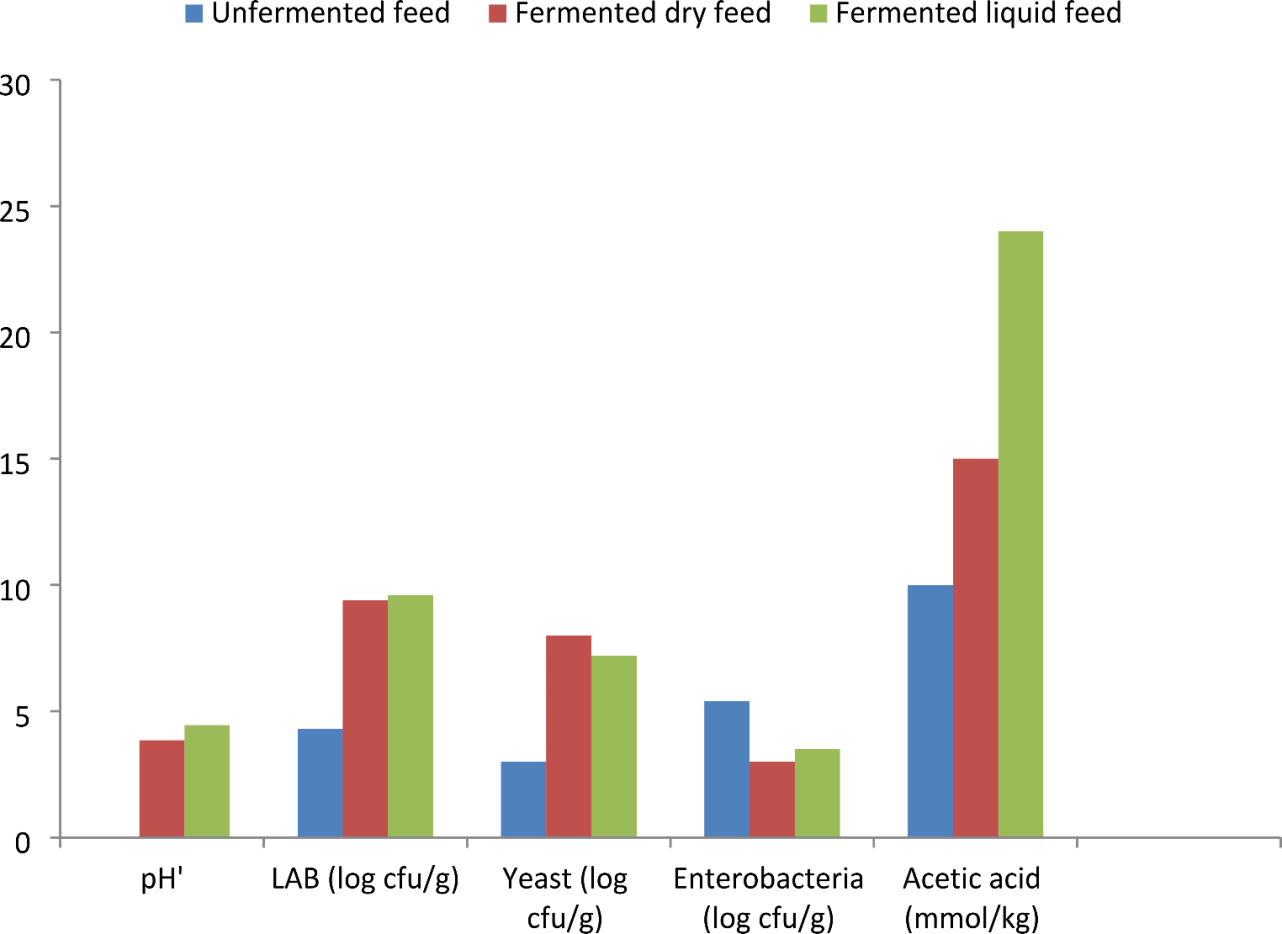
Effect of fermentation on aspects of chemical and microbial characteristics of dry and liquid feed. Adapted from Sugiharto and Ranjitkar (2019)

Fermentation has been found to enhance digestibility, nutritional, and microbial composition of feedstuffs (Chukwukaelo et al. 2018; Sugiharto and Ranjitkar 2019; Shi et al. 2021). Ranjitkar and Engberg (2016) found an increased lactic and acetic acid content in maize kernel fermented for 56 days. In the same study, the authors found a 24% reduction in pH, a 50% decrease in mould and enterobacteria growth, and a 9% increase in LAB growth. In a similar study, Chukwukaelo et al. (2018) also discovered that SSF of a mixture of grated cassava roots and palm kernel cake increased its total ash and crude protein content by 0.23 and 50.46%, respectively, and reduced its ether extract level by 23%. These support the earlier reports of others (Canibe et al. 2007; Sugiharto and Ranjitkar 2019) that fermentation promotes the growth of LAB, production of organic acids, lower pH, and inhibits the growth of pathogenic microorganisms (Fig. 1). This positions fermented feed as a safe feed with the potential to reduce the buildup of mycotoxins in feedstuffs and finished feeds, as well as improve chicken’s intestinal health and wellbeing of chickens (Ranjitkar and Engberg 2016; Aladi et al. 2017; Yang et al. 2018). This also indicates that fermentation microbes degrade mycotoxins into non-toxic compounds. Fermented feed materials also contain biogenic amines (histamine, cadaverine and others) and acetic acids, which may hamper feed palatability and performance of chickens offered such feed (Sugiharto and Ranjitkar 2019). Likewise, Londero et al. (2014) observed that adding whey fermented for 24 h at 20^o^C to the chicken diet protects it against fungal contamination. Apart from reduced microbial contamination, Sugiharto and Ranjitkar (2019) found that metabolites such as organic acids and bacteriocins generated by LAB during fermentation may extend the shelf life of fermented feedstuffs and finished feed. The incorporation of LAB, organic acids, or exogenous enzymes into feed materials before fermentation has been shown to enhance the nutritional properties and palatability of finished feed (Canibe and Jensen 2021). Similarly, Hassaan et al. (2015) found that yeast fermentation increased the content of crude protein, ash, ether extract, and total amino acids in soybean meal while decreasing the crude fibre, trypsin inhibitor, and phytate levels. The increase in crude protein in fermented soybean meal compared to unfermented type could be attributed to increased production of microbial cell mass. In converse, the decline in crude fibre level in fermented soybean meal, compared with unfermented soybean meal could be due to the production and release of varieties of enzymes by fermentation microbes that breakdown fibre, as previously documented in the literature (Belewu and Sam 2010; Hassaan et al. 2015; Shi et al. 2021).

## Biocomponent profiles of fermented novel feed materials

Low protein content, high fibre level, reduced digestibility, and the presence of ANFs limit the intake of novel feedstuffs in chicken feed (Ogbuewu et al. 2015; Ogbuewu and Mbajiorgu 2023c, d). The potential of fermentation in enhancing the feeding quality of novel feedstuffs in poultry nutrition has been highlighted (Aladi et al. 2017; Chukwukaelo et al. 2018; Sugiharto and Ranjitkar 2019). Shi et al. (2021) found that SSF of *Moringa oleifera* leaf meal (MLM) increased its total ash, crude protein, and total amino acid content, and reduced its phytic acid, crude fibre, and tannins. In a similar study, Wang et al. (2018) reported that fermentation improved the nutritional quality and digestibility of MLM. These findings are consistent with the results of other investigators (Londero et al. 2014; Aladi et al. 2017; Sugiharto and Ranjitkar 2019), who reported that fermentation improves the nutritional characteristics of feed resources by improving their fibre, ether extract, crude protein, amino acid content, protein solubility, and vitamin bioavailability. Fermentation has been demonstrated to reduce the content of ANFs, such as cyanogenic glycosides, glucosinolates, lectins, prosopine, tannins, trypsin inhibitors, haemagglutinins, and phytate in alternative feedstuffs (Chukwukaelo et al. 2018; Sugiharto and Ranjitkar 2019). In a similar study, Sokrab et al. (2014) showed that phytase produced by microbes during fermentation may assist in the breakdown of phytates bound in the fibre matrix of leaf meals, resulting in decreased phytate levels in fermented products. Furthermore, enzymes synthesised by microorganisms during fermentation processes detoxify ANFs and reduce the level of heavy metals in the feed materials (Prasad and Freitas 2000; Tefera et al. 2014; Kiczorowski et al. 2022). The incorporation of essential oils derived from fermented tropical leaves into the chicken diet as performance-enhancing agents has been evaluated (Puvaca et al. 2022). Solid-state fermentation of cinnamon leaves reduced the lignocellulosic biomass levels and increased the oil yield (Abduh et al. 2021). The observation could be ascribed to the ability of fermentation microbes to degrade cell wall components of cinnamon, resulting in a larger surface area so that the diffusion rate is higher and consequently leads to a higher oil yield.

## Tropical leaf meals with potential for application in the poultry industry

Tropical climates are endowed with plants whose leaves remain evergreen at critical times of the year (Achonwa et al. 2019) and yield large amounts of leaf biomass with potential for commercial production (Table 1). Several of these plants are presently utilised by humans for a variety of purposes, including forage, fodder, food, fuel, fibre, oil, herbs, and among others. Many tropical plant leaves are rich in nutrients and possess several medicinal values (Sebola et al. 2015; Achonwa et al. 2019; Ogbuewu and Mbajiorgu 2019). Moringa leaf, one of such tropical leaves, belongs to the family Moringaceae. Nutritional analysis revealed that MLM is high in nutrients, including crude protein, fibre, carbohydrates, ether extract, and ash (Falowo et al. 2018; Modisaojang-Mojanaja et al. 2019). According to Falowo et al. (2018), MLM contains 16–19 amino acids, of which ten are essential amino acids. In addition, MLM contains the following beneficial bioactive compounds: terpenes, flavonoids, polyphenols, hexyl 3-methylbutanoate, diethyl phthalate, methyl palmitate, and dimethyl-propanedioic acid (Falowo et al. 2018). Aside from the bioactive compounds, MLM has high levels of ANFs such as oxalate and hydrogen cyanides (Shih et al. 2011; Falowo et al. 2018), which may impair digestion and nutrient uptake in chickens. Research has shown that oxalate chelates zinc, iron, and calcium and therefore diminishes their bioavailability. A high level of oxalate reduces copper bioavailability by binding it to form insoluble salt (Umaru et al. 2007).

**Table 1:** Botanical description of some tropical leaf meals utilised in poultry production

Scientific name	Common/English name	Family
*Moringa oleifera*	Drumstick tree, horseradish tree	Moringaceae
*Musa spp*	Banana	*Musaceae*
*Carica papaya*	Pawpaw	*Caricaceae*
*Mangifera indica*	Mango	*Anacardiaceae*
*Mucuna pruriens*	Velvet bean	*Fabaceae*
*Microdesmis puberula*	Microdesmis	*Pandaceae*
*Dialium guineense*	Black velvet tamarind	*Fabaceae.*
*Elaeis guineensis*	Oil palm	*Arecaceae*
*Aspilia africana*	Haemorrhage plant	*Compositae*
*Chromolaena odorata*	Siam weed, Christmas bush, Devil weed	*Asteraceae*
*Manihot spp*	Cassava	*Euphorbiaceae*
*Amaranthus spp*	African spinach	*Amaranthaceae*
*Gongronema latifolium*	Bush buck	*Asclepiadaceae*
*Azadirachta indica*	Neem	*Meliaceae*
*Tridax procumbens*	Coat buttons	*Asteraceae*
*Ficus microcarpa*	Chinese banyan, Malayan banyan	*Moraceae*
*Albizia spp*	Lebbeck	*Fabaceae*
*Ginkgo biloba*	Ginkgo	*Ginkgoaceae*
*Olea europaea*	Olive	*Oleacea*
*Corchorus olitorius*	Jute	*Malvaceae*
*Morinda citrifolia*	Noni	*Rubiaceae*
*Sauropus androgynus*	Sweet leaf	*Phyllanthaceae*
*‎Opuntia ficus-indica*	Prickly pear	*Cactaceae*
*Annona muricata*	Soursop	*Annonaceae*
*Tapinanthus bangwensis*	African mistletoe	*Loranthaceae*
*Pentaclethra macrophylla*	African oil bean	*Fabaceae*
*Ginkgo biloba*	Ginkgo	*Ginkgoaceae*

*Source* Syahruddin et al. (2011); Ifesan et al. (2014); Mandey et al. (2015); Santoso et al. 2015a, b, 2018); Sebola et al. (2015); Ogbuewu et al. (2015); Zhou et al. (2015); Rasaq (2017); Siaboc (2018); Xie et al. (2016); Manihuruk et al. (2018); Achonwa et al. (2019); Modisaojang-Mojanaja et al. (2019); Ogbuewu and Mbajiorgu 2019, 2023a; Okoli et al. (2019); Sugiharto and Ranjitkar (2019); Mat et al. (2020); Sugiharto et al. (2020); Sugiharto (2021); Shi et al. (2021); Niu et al. (2022)

*Ginkgo biloba* is an important tree with nutritional and medicinal benefits (Singh et al. 2008). Ginkgo leaves contain polysaccharides, minerals, essential oils, and amino acids (Yu et al. 2021), which are vital for chicken health (Cao et al. 2012; Niu et al. 2017). *G. biloba* leaf meal (GLM) contains terpenoids, flavonoid glycosides, organic acids, and other beneficial compounds that have been shown to have pharmacological effects, such as antioxidant and antimicrobial properties (van Beek and Montoro 2009; Liu et al. 2021). Research indicates that GLM has other pharmacological effects, including anticancer, antifungal, and anti-inflammatory properties (Sati and Joshi 2011; Liu et al. 2013). However, the presence of ginkgotoxin and other toxic compounds in fresh ginkgo leaves limits their use in chicken nutrition (Nwosu et al. 2018). In a similar study, Princewill-Ogbonna et al. (2019) and Ali et al. (2020) found that *Magnifera indica* leaf meal (MILM) contains several essential nutrients, including vitamins, minerals, and amino acid effect, with the potential to enhance chicken productivity. The authors further reported that MILM contains phytonutrients, such as essential oils, saponins, phenols, and flavonoids, that may have antimicrobial and growth promoting effects.

*Microdesmis puberula* is a browse plant whose leaf meal (MPLM) contains a wide range of phytochemicals (Udo et al. 2017). Research has shown that *M. puberula* leaf meal (MPLM) is high in important nutrients required to support the overall health and productivity in chickens (Esonu et al. 2003, 2004; Udo et al. 2017; Okeke et al. 2023). In a similar study, Udo et al. (2017), noticed that MPLM are low in ANFs, such as hydrogen cyanides, oxalates, and phytic acids, which have been shown to impair digestion and nutrient utilisation when consumed by chickens in large amounts (Yamamoto et al. 2010). *Morinda citrifolia* (Noni) is a fruit-bearing tree native to Southeast Asia, and its leaf meal is high in amino acids, anthraquinones, fatty acids, flavonoids, iridoids, lignans, polysaccharides, sterols, sugars, and terpenoids. Morindone, morindion, morindanidrine, alizarin, 3-hydroxy morindone, rubiadine, and lucidin are the active compounds in *M. citrifolia* that give it its therapeutic effects (Ali et al. 2016). Aside from the aforementioned essential bioactive substances, mature *M. citrifolia* leaves are high in fibre and moderate in ANFs, which may restrict their application in practical poultry diets.

## Nutrient composition of fermented TLMs

Plant leaves contain an appreciable level of protein, amino acids, minerals, fatty acids, and vitamins that are vital for poultry production (Ogbuewu et al. 2015; Modisaojang-Mojanaja et al. 2019). However, the use of leaf meals in chicken feed is constrained by their high fibre value and varying concentrations of ANFs (Ogbuewu et al. 2015; Achonwa et al. 2019), which may limit their use in chicken feed at high inclusion levels. Fermentation can help reduce the concentrations of fibre and ANFs in leaf meals (Sugiharto and Ranjitkar 2019). The effect of fermentation on nutrient and ANF characteristics of TLMs is shown in Table 2. According to Shi et al. (2021) fermentation increased the protein content of MLM by 44% while reducing the fibre level by 70% when compared to unfermented MLM. Rasaq (2017) also noted similar results in MPLM. The enhanced protein content of TLMs due to fermentation may be ascribed to increased microbial biomass, implying that fermented leaf meals could serve as a good protein source for chickens. The authors also observed that fermentation increased the amino acid content of MLM, except for lysine and glutamic acid, which decreased. One possible explanation for this observation is that microbes involved in fermentation may have used glutamic acid and lysine for their metabolic activity. Shi et al. (2021) also observed that fermentation increased the concentrations of essential amino acids, non-essential amino acids, and total amino acids (TAA) in fermented MLM (fMLM) compared to the unfermented MLM.

**Table 2:** Effect of fermentation on nutrient and anti-nutrient factor characteristics in TLMs

Parameters (%)	BLM		MLM		Mucuna leaf meal		CPLM		MILM		VALM	
	UF	F	UF	F	UF	F	UF	F	UF	F	UF	FF
Dry matter	33.95	29.75			97.0	93.40	87.6	86.7	93.44	92.66	96.73	94.20
CP	11.52	16.70	21.12	30.19	24.50	24.94	26.7	29.1	6.16	8.64	23.51	21.92
CF	27.73	31.30	27.35	25.17	20.71	25.27	34.2	18.5	16.4	11.7	10.97	14.55
EE	4.15	4.64	4.68	4.42	4.40	4.20	11.9	5.96	8.22	9.78	6.37	7.39
Ash	20.52	12.20	8.05	10.13	2.10	2.90	13.8	10.8	29.9	32.30	12.95	23.28
Calcium	0.14	0.11	0.90	1.14								
Iron	0.03	0.03										
TAA			12.12	14.11								
NDF			51.62	57.32								
ADF			37.93	34.95								
Hemicellulose,			13.57	22.15								
Cellulose			25.37	21.50								
Lignin			10.44	10.55								
Phytate			5.94	3.61					2.44	2.43		
Tannins			8.39	3.63					0.08	0.05		
References	Mat et al. (2020)		Shi et al. (2021)		Woke et al. (2013)		Sugiharto et al. (2020)		Ojokoh (2007)		Ifesan et al. (2014)	

UF – Unfermented; F – Fermented; CP – Crude protein; CF – Crude fibre; EE – Ether extract; TAA – Total amino acids; NDF – Neutral detergent fibre; ADF – Acid detergent fire; BLM – Banana leaf meal; MLM – *Moringa oleifera* leaf meal; VALM – *Vernonia amygdalina* leaf meal; CPLM – *Carica papaya* leaf meal; MILM – *Magnifera indica* leaf meal

Apart from the above-mentioned nutritional quality of tropical leaf meals, Mat et al. (2020) found that fermentation increased the total ash and crude protein content of banana leaves while decreasing their ether extract and crude fibre contents. This finding supports Chukwukaelo et al. (2018) and Shi et al. (2021), who discovered that fermentation decreased ether extract and crude fibre levels in MLM, indicating that fermentation microbes used fat for their metabolic activity. The decline in fibre may be attributed to the activity of fibre-degrading enzymes produced by microorganisms during fermentation. Apart from enhancing the nutritional quality of feed and feed ingredients, the fermentation process improved the safety of fermented feed by producing organic acids that reduced the pH of the product, encouraging the proliferation of fermentation bacteria (Canibe et al. 2007; Sugiharto and Ranjitkar 2019). Another explanation of this effect involves a decrease in pH caused by lactic acid and, consequently, a reduction in the activity of starch hydrolysing enzymes (Knez et al. 2023).

## Phytochemicals in fermented tropical leaf meals

Phytochemicals are chemicals produced by plants through primary or secondary metabolism. They generally have biological activity in the plant host and play a role in plant growth or defence against competitors, pathogens, or predators. Phenols are the main bioactive phytochemicals found in fermented TLMs (Shi et al. 2021; Sugiharto 2021), but their composition differs depending on the environment, post-harvest storage, soil type, plant genetics, processing methods, and other factors (Ndubuaku et al. 2014). Phenols assist to protect the body against diseases by scavenging free radicals, chelating metals, releasing antioxidative enzymes, and reducing peroxides. Fermentation has been found to increase the levels of phenols and flavonoids in TLMs (Table 3). This observed increase in levels of phenols and flavonoids may be attributed to the activities of numerous extracellular enzymes produced by fermentation microbes, which release the flavonoids and phenols bound in the fibre matrix of leaf meals (Dulf et al. 2016; Dey et al. 2016).

**Table 3:** Effect of fermentation on phytochemical composition and selected nutrients on tropical leaf meals

Parameters (mg/g)	GLM			GLM			GLM			MLM		
	UF	F	Increase	UF	F	Increase	UF	F	Increase	UF	F	Increase
Total flavonoids (mg/g)	9.7	9.4	-0.3	6.6	7.5	0.90	26.45	24.67	-1.78	5.50	5.00	-0.50
Polysaccharides (g/kg)	4.37	6.51	2.24	4.37	6.42	2.05	4.37	6.46	2.09			
Proteins (g/kg)	103.7	179.9	76.2	21.06	33.43	12.37	136.4	270.1	133.7			
Total amino acids (g/kg)	76.33	92.55	16.22	15.65	20.51	4.86	95.98	135.11	39.13			
Total ginkgolic acid (g/kg)				1.66	0.054	-1.61	1.67	0.06	-1.61			
Protease activity (Unit/g)				100	260	160						
β-glucosidase activity (Unit/g)				0.4	3.5	3.1						
Phenols										40.00	23.00	-17
Alkaloids										17.33	12.33	-5
Saponins										9.83	7.50	-2.33
Terpenoids										20.00	25.00	5
References	Cao et al. (2012)			Yu et al. (2015)			Zhang et al. (2012)			Ijarotimi et al. (2013)		

UF – Unfermented; F – Fermented; GLM – Ginkgo leaf meal; MLM – *Moringa oleifera* leaf meal

Tropical leaves also contain carotenoids, which are classified as terpenoids (Ali et al. 2013). Carotenoids are divided into carotenes and xanthophylls. In nature, 𝛽-carotene is the most abundant type of carotene. Among the xanthophylls, lutein is typically detected in tropical leaves. A detailed review by Falowo et al. (2018) categorised the phytochemicals in TLM into hydrocarbon, alcohol, terpenes, ketones, fatty acids, aldehydes, and others using GC–MS or HS-SPME methods. Some of the detected phytochemicals in TLMs have antipyretic, antioxidant, anticancer, antimicrobial, anti-inflammatory, antifungal, anti-ulcerative, and antipyretic activities (Falowo et al. 2018). The predominant ANFs in tropical leaves are tannins, saponins, oxalates, phytic acid, trypsin inhibitors, and glucosinolates (Rasaq 2017; Achonwa et al. 2019). These ANFs, though not necessarily harmful, may interfere with digestion and assimilation of some minerals when taken in large amounts. According to Rasaq (2017) found that fermentation reduced the phytate content in fermented TLMs. This finding agrees with Shi et al. (2021), who found that fermentation has the capacity to reduce the amount of ANFs in feed materials. Though the exact mechanism by which fermentation reduces phytate levels in TLMs is not clear, it is possible that phytase enzymes produced by microbes during fermentation breakdown phytates to release inorganic phosphate and inositol.

### Effect of fermented TLMs on broiler chicken performance

The use of fermented leaf meals in livestock diets is attributed to their richness in fibre and moderate concentration of ANFs. Fibre is an essential component of animal feed and has been found to aid gut peristalsis and reduce the transit time of digesta in the gastrointestinal tract (Jha and Mishra 2021). The authors also noticed that soluble fibres increase digesta viscosity, which in turn decreases digestibility and uptake of nutrients in the chicken, leading to poor feed intake and body weight gain (BWG). However, the inclusion of large amounts of fibre in chicken feed may dilute nutrient density and reduce digestibility of feed mixtures. Tropical leaves are high in fibre and cannot be added to chicken feed in a large amount because of the inability of chickens to produce enzymes that breakdown and digest fibre. However, evidence exists that fermentation breaks down fibrous biomass into its basic units (Chukwukaelo et al. 2018; Sugiharto and Ranjitkar 2019). As presented in Table 4, broiler chickens fed a commercial diet without fMLM substitution had significantly higher feed intake, superior BWG, and feed conversion ratio (FCR) than broilers fed a commercial diet substituted with fMLM at 2.5, 5.0 and 7.5% (Zulfan et al. 2021). The observed poor performance suggests that substituting a commercial diet with fMLM at 2.5, 5.0, and 7.5% diluted the nutrient level of the diet. This finding agrees with Sebola et al. (2015) and Modisaojang-Mojanaja et al. (2019), who demonstrated that dilutions of finished feed with TLMs beyond the optimal inclusion levels will ultimately result in lower feed intake, possibly due to an increase in digesta viscosity and a longer retention time of the digesta in the gastrointestinal tract. This explains the decline in feed intake to incremental substitution levels of fMLM as reported by Zulfan et al. (2021).

**Table 4:** Effect of dietary fermented tropical leaf meals on body weight gain (BWG) of broiler chickens

References	Broiler strain	Study duration (days)	Leaf type	Inclusion level	Control BWG (g)	Treatment mean BWG (g)	Main findings
Cao et al. (2012)	Arbor Acres	42	*Aspergillus niger*- fermented *Ginkgo biloba* leaf meal (fGLM)	Starter phase: 0, 0.2, 0.35, and 0.5% Finisher phase: 0, 0.4, 0.70, and 1.0%	2.39	2.42	In comparison with the control, the addition of *Aspergillus niger*-fGLM in broiler diets increased BWG by 1.35%.
Zhang et al. (2012)	Arbor Acres	42	*Aspergillus niger- fermented Ginkgo biloba leaf meal (fGLM)*	Starter phase: 0.2, 0.35, and 0.5%. Finisher phase: 0.4, 0.70, and 1.0%	50.13	50.8	The addition of *A. niger*-fGLM in broiler diets increased BWG by 1.34% when compared with the control.
Syahruddin et al. (2013)	Lohmann	56	*Trichoderma harzianum* fermented *Sauropus androgynus* leaf meal (fSLM)	0, 2, 4, 6, 8, 10, 12, and 14%	46.54	44.35	Incorporation of *T. harzianum* fSLM in broiler chicken diets at 2, 4, 6, 8, 10, 12, and 14% reduced BWG by 4.71%
Yu et al. (2015)	Arbor Acres	42	*Bacillus spp*. fermented *Ginkgo biloba* leaf meal (fGLM)	0 and 0.35%	54.60	57.5	Dietary *B. natto* and *B. licheniform* fermented fGLM at 0.35% increased BWG in broiler chickens by 5.31%%.
Zhang et al. (2015)	Arbor Acres	42	Fermented *Ginkgo biloba* leaf meal (fGLM)	0 and 0.5%	52.23	53.23	Dietary fermented fGLM at 0.5% increased BWG in broiler chickens by 6.18%.
Niu et al. (2017)	Arbor Acres	42	Fermented *Ginkgo biloba* leaf meal (fGLM)	0, 1.5, 2.5, 3.5, 4.5, and 5.5 g/kg	53.60	54.98	Results showed that addition of fGLM in broiler chicken rations at 3, 6 and 9% for 42 days increased their BWG by 3.14%.
Niu et al. (2019)	Arbor Acres	42	Fermented *Ginkgo biloba* leaf meal (fGLM)	0, 1.5, 2.5, 3.5, 4.5 and 5.5 g/kg	2.29	2.36	The study indicated that addition of fGLM in broiler chicken rations at 3, 6 and 9% for 42 days increased their BWG by 12%.
Zhang et al. (2020)	Arbor Acres	42	Fermented *Ginkgo biloba* leaf meal (fGLM)	0, 1, 3 and 6 g/kg	2.09	2.17	Results revealed that fGLM increased BWG in broiler chickens by 3.74%.
Ding et al. (2021)	Yellow feathered	56	Fermented mulberry leaf powder (fMLP)	0, 3, 6 and 9%	28.70	32.07	Dietary supplementation of fMLP in Yellow feathered broiler chicken diets at 0.5, 2.5, 3.5, 4.5, and 5.5 g/kg feed for 42 days increased BWG by 2.57%.
Zulfan et al. (2021)	CP 707	35	Fermented *Moringa oleifera* leaf meal (fMLM)	0, 2.5, 5.0 and 7.5%	11.51	10.76	Inclusion of fMLM in broiler chicken diets at 2.5, 5.0 and 7.5% reduced BWG by 6.52%
Xie et al. (2021)	Arbor Acres	42	Fermented *Chenopodium album* leaf meal (fCALM)	0, 2, 4 and 8 g/kg	53.87	56.56	Results showed that dietary of fCALM increased BWG in broiler chickens by 5%.

Mandey et al. (2015) found improved growth performance in broilers fed 10% fermented banana leaf meal. A similar trend has been reported in broilers fed fermented Ginkgo leaf meal (fGLM) (Yu et al. 2015; Kim et al. 2017; Niu et al. 2019). Likewise, Siaboc (2018) found enhanced growth performance in broilers on dietary fermented jute (*Corchorus olitorius*) leaf meal supplementation. Xie et al. (2016) also found enhanced growth traits in broilers fed fermented olive leaf meal residue. However, contradictory evidence may also exist in the literature (Zhang et al. 2012). The inclusion of more than 1% of fGLM in broiler diets reduced BWG and FCR (Zhang et al. 2012). This implies that broilers’ response to fermented TLMs depends on the type and quantity of TLMs added to the feed. The presence of residual ANFs in fGLM, which may not have been completely removed by fermentation, may have led to the poor growth performance in broilers fed diets having high inclusion levels of fGLM (Santoso et al. 2015a).

## Impact of fermented TLMs on carcass and meat quality of broilers

Broilers are specifically bred to produce high-quality meat in a short period. Consumer preference for quality is currently on the increase. Farmers are responding to this by producing meat low in fat, saturated fatty acids (SFA), and cholesterol, and high in polyunsaturated fatty acids (PUFA) through dietary manipulations (Sugiharto and Ranjitkar 2019). Studies suggest that inclusion of fermented leaf meals of *Morinda citrifolia*, *M. oleifera*, and *S. androgynus* in broiler diets had no influence on dressing percentages (Syahruddin et al. 2011; Santoso et al. 2015a; Manihuruk et al. 2018). Increased abdominal fat deposition in broiler chickens suggests poor dietary energy use efficiency. The addition of fermented leaf meals to broiler chicken diets reduced meat cholesterol, fat and total fatty acid levels, and abdominal fat content (Zhang et al. 2020; Sugiharto et al. 2020), indicating an improvement in dietary energy use efficiency. This observation is consistent with the earlier results of Syahruddin et al. (2013) and Sugiharto (2021), who found reduced cholesterol content in meats of broilers fed fermented TLMs. The observed reduction might be related to the ability of LAB and bioactive compounds present in fermented TLMs to change the lipid metabolic pathway, thus inhibiting cholesterol production and fat deposition in the muscle (meat) and abdomen (Sugiharto et al. 2020; Sugiharto 2021). In humans, SFAs are linked to cardiovascular diseases, while PUFAs have health-promoting effects. As a result, consumers prefer broiler meats that are rich in PUFA and low in SFA. Cao et al. (2012) reported that dietary fGLM reduced the levels of palmitic acid (C16:0) and octadecanoic acid (C18:0) and increased the levels of arachidonic acid (C20:4), linoleic acid (C18:2) and α-linolenic acid (C18:3) in meats. Similarly, Kim et al. (2017) found that inclusion of fGLM in broiler diets at 5 and 10% decreased the levels of SFA and monounsaturated fatty acids (MUFA) in thigh and breast muscles, while increasing the levels of n-3 PUFA. Sugiharto et al. (2020) found improved levels of PUFA and reduced levels of MUFA in the breast muscle of broilers fed fMLM but did not affect SFA. In a similar feeding study, Santoso et al. (2015b) found an increased ratio of saturated to unsaturated fatty acids, β-carotene, and vitamin E and A, in the breast muscle of broilers fed fSLM. The increased ratio of saturated to unsaturated fatty acids contradicted the recent findings of Sugiharto (2021). This discrepancy could be attributed to differences in composition of fermented TLMs, the microorganisms involved in fermentation, diet composition, and other factors.

## Influence of fermented TLMs on gut histomorphology of chickens

There is an expanding body of knowledge supporting the use of fermented plant products to improve the physiological wellbeing of broilers (Ogbuewu and Mbajiorgu 2023 c, d). Niu et al. (2019) reported that fGLM supplementation increased the weight of duodenum in broilers, suggesting an improvement in the absorptive capacity of the small intestine. In a recent study, Niu et al. (2022) observed that supplementation of 5% fermented *B. papyrifera* in a layer diet did not influence intestinal morphology and microbiota composition in chickens, implying the safety of its use in laying hens. It is known that intestinal mucosa inhibits the passage of toxic substances from the lumen, stops the invasion of pathogenic bacteria, and plays a major role in the feed digestion and nutrient uptake. The villi height (VH), crypt depth (CD), and VH/CD ratio are the key histological indices used to assess the absorptive capacity of the small intestine (Niu et al. 2019, 2022). According to Tian et al. (2022), layers fed a diet having 500 mg/kg lactobacillus fermented herbs (LFH) had superior absorptive capacity of the small intestine when compared to the control group. Also, the mRNA expression of tight junctions showed that 500 mg/kg of LFH supplementation upregulated the expression of Occludin and ZO-1 genes in the jejunum of hens compared to the birds offered a control diet. In addition, the authors reported that dietary LFH supplementation influenced caecal microbiota composition in laying hens.

### Effect of fermented tropical leaf meals on physiological parameters of chickens

Blood and its constituents provide accurate data for the assessment of the health status of farm animals. Reports on the health status of broilers fed fSLM at 50 g/kg feed had increased hematocrits (Htc), white blood cell (WBC), and red blood cell (RBC) count (Santoso et al. 2015a). The mechanism by which fermented feed improved blood values in chickens is not clear; however, the observed increase in blood values of broilers fed fermented TLMs may be ascribed to improved quality of protein contained in the diet as a positive correlation exists between RBC and dietary protein (Etuk et al. 2014; Ogbuewu et al. 2015). In a similar experiment, Kim et al. (2017) found increased serum immunoglobulin A and G levels, and cytokine secretion in Ross 308 broilers fed a basal diet supplemented with fGLM at 5 and 10% for 35 days. This improvement may be related to increased production of functional bioactive compounds such as terpenes and flavonoids during fermentation of GLM by LAB. Improved immune responses in animals other than broilers fed 10% fGLM have also been reported (Zhou et al. 2015). This could be due to the conversion protein content in fGLM to peptides and amino acids shown to have immunomodulatory properties (Sachindra and Bhaskar 2008). Santoso et al. (2018) reported that inclusion of up to 5% fermented mixture of *S. androgynus* and bay leaf had no adverse effect on RBC and WBC in broilers. This contrasted the findings of other researchers (Santoso et al. 2015a; Zhou et al. 2015; Kim et al. 2017). The observed disparity may be attributed to differences in inclusion levels and the nature of leaves used.

The incorporation of fermented TLMs into chicken diets has been shown to affect lipid metabolism (Ogbuewu et al. 2015; Sugiharto 2021). Zhang et al. (2020) show that GLM supplementation reduced serum cholesterol levels in broilers. Likewise, feeding broilers fermented blend of *S. androgynus* and bay leaf reduced serum cholesterol, low density lipoprotein (LDL), triglycerides, and increased serum high-density lipoprotein (HDL) levels (Santoso et al. 2018). Inclusion of fermented herbal products in chicken feed increased serum HDL and reduced cholesterol, LDL, and triglycerides levels in broilers (Lee et al. 2019; Sugiharto 2021; Niu et al. 2023), suggesting possible hypocholesterolemic and lipolytic activities of fermented TLMs. Similar results were also observed by adding fGLM in the diets of laying hens (Zhao et al. 2013). The observed decrease in serum lipid content in broilers fed fermented TLMs could be attributed to the action of flavonoids in the fermented leaves that have cholesterol-lowering potential (Kim et al. 2004; Ting et al. 2011). Flavonoids may also decrease serum lipid levels in broilers by upregulating hepatic β-oxidation and downregulating lipid biosynthesis. In converse, Santoso et al. (2015b) stated that dietary fSLM had no impact on serum lipid profiles in broilers. The variations differences could be due to differences in diet composition, microbes involved in fermentation process, and the types of leaves used.

Broilers are prone to stress as a result of their management system, which can result in oxidative damage and poor performance. The use of natural products with proven antioxidant properties in animal production to enhance productivity has been stated (Sugiharto 2021). Increased antioxidative status of broilers fed fermented TLMs has been highlighted as marked by increased levels of serum vitamin E and antioxidative biomarkers such as superoxide dismutase (SOD) and glutathione (Cao et al. 2012; Zhang et al. 2015; Xie et al. 2016). In a similar study, Zhang et al. (2020) discovered that fermented TLMs improved the antioxidant status of broilers, as evidenced by increased serum levels of glutathione, vitamin E, glutathione peroxidase (GPx) and SOD and a reduction in serum malondialdehyde level. This was accomplished by upregulating the expression of antioxidant enzyme genes by fermented TLM (Zhang et al. 2020). Further to this, Niu et al. (2019) stated that fermented TLMs increased antioxidant activity in chickens, as evidenced by increased concentrations of total antioxidant capacity, GPx, and catalase (CAT) in the small intestine.

Apart from the absorption of nutrients, the intestine also plays an important role in the immune system of broilers (Sugiharto 2021). The local and systemic immune systems of broilers can be modified by the intestinal immune system. Zhang et al. (2015) found fermented TLMs increased proliferation of LAB and decreased *E. coli* and Salmonella counts in the small intestine of broilers fed varying levels of fermented leaves. The reduction in *E. coli* and Salmonella populations in the intestines of broilers fed fermented TLMs may be connected to the antibacterial properties of LAB and their metabolites, such as organic acids and bacteriocins, which are in abundance in fermented TLMs (Sugiharto and Ranjitkar 2019). Currently, there is a lack of published data on the mechanism by which fermented leaves modulate gut microbiota composition and immune response of chickens; as a result, future research should focus on this area.

### Influence of fermented TLMs on productive indices of laying hens

Egg production and quality are indices used to determine productivity of laying hens, and increased egg production is one of the key parameters to maximise profitability. Table 5 shows the positive influence of fermented TLMs on egg production and quality indices in laying hens. Antara et al. (2019) observed that inclusion of fermented herbal extract has no effect on feed intake in layers, but significantly increased egg weight. Siti and Bidura (2018) noticed that oral administration of fermented *M. oleifera* leaf extract (fMLE) at 2, 4, and 6 mL to laying hens increased egg production and yolk colour. On the same hand, layers fed fermented herbal products had increased egg production and egg quality and reduced FCR, serum cholesterol, triglyceride, and LDL (Zhao et al. 2013; Lee et al. 2019; Kothari et al. 2021). These findings agree with the results of others (Lin et al. 2017; Siti et al. 2017) in layers fed TLMs. The enhanced yolk colour in layers fed TLMs can be explained by varied carotenoid content in tropical leaves (Zhao et al. 2013; Cayan and Erener 2015; Du et al. 2020). The bioactive compounds in fermented TLMs may have improved laying performance via one or a combination of the following mechanisms: increased activity of digestive enzymes, direct nutritional effect, inhibition of gut pathogens via competitive exclusion, as well as improvement in the absorptive capacity of the small intestines (Grashorn 2010).

**Table 5:** Effect of fermented TLMs on performance of laying hens

References	Layer strain	Duration of feeding	Treatments	Main results
Zhao et al. (2013)	49-weeks-old Lohmann Brown	8 weeks	0% fGLM, 0.5% *Candida utilis* fGLM, 0.5% *Aspergillus niger* fGLM, and their combined fermentation	Results show that inclusion of GLM fermented with *C. utilis*, *A. niger* and their mixture in the diet of laying hens aged 49 weeks improved laying rate and FCR. Results also show that inclusion of fGLM at 0.5% decreased cracked-egg rate, and egg yolk cholesterol content, but had no effect on egg mass, egg weight, and feed intake.
Park et al. (2016)	Hy-Line brown	5 weeks	T0 = basal diet without fermented *Gynura procumbens* and *Rehmannia glutinosa*, T1 = FPFA1 (basal diet + 0.5% *G. procumbens* and *R. glutinosa*, T2 = basal diet + 1% *G. procumbens* and *R. glutinosa*, and T3 = basal diet + 2% *G. procumbens* and *R. glutinosa*.	Supplementation of fermented blend of *G. procumbens* and *R. glutinosa* at 1 and 2% improved egg production, egg weight, albumen height, and Haugh unit in layers when compared to layers fed diet without fermented blend of *G. procumbens* and *R. glutinosa*. Fermentation microbes (probiotics) and bioactive compounds in fermented *G. procumbens* and *R. glutinosa* may explain the improved laying performance.
Syahruddin et al. (2016)	20-weeks-old Native laying hens		0, 5.87, 11.74, 17.61, 23.48, and 29.35% fermented rubber leaf and seed meals.	Results showed that inclusion of up to 29.35% of fermented rubber leaf and seed meals had no effect on feed intake, FCR and egg production in native laying hens. Results also indicate that inclusion of up to 29.35% of fermented rubber leaf and seed meals in laying diets had no significant effect on egg weight, eggshell thickness, and yolk colour score.
Antara et al. (2019)	70 weeks old indigenous laying hens	nr	T1 = drinking without *Moringa oleifera* leaf extract (MLE); T2 = drinking water with 2% MLE; and drinking water with 4% MLE.	Result revealed that aqueous MLE improved egg weight and egg production compared to layers on control treatments. Higher egg mass was recorded in birds offered MLE at 2 and 4% than the control diet. MLE improved FCR and egg cholesterol in laying hens.
Lee et al. (2019)	40-weeks-old Hy-Line Brown layers	6 weeks	Basal diet (CON), basal diet (BD) + 0.5% *Lactobacillus plantarum*, BD + 0.5% non-fermented *A. annua* leaf meal, and BD + 0.5% fermented *A. annua* leaf meal (fALM).	Layers fed a basal diet supplemented with 0.5% fALM had better eggshell colour and Haugh unit than those offered control diet. However, fALM had no effect on yolk colour, eggshell strength, and thickness. It was observed that during egg storage at 18 °C, the Haugh unit and malondialdehyde of eggs from layers fed basal diet supplemented with 0.5% fALM, 0.5% *L. plantarum* and 0.5% non-fermented ALM was improved when compared to those layers that received basal diet only.
Bidura et al. (2021)	40-week-old laying hens	Not reported	Dietary treatments were: 0% fermented *Dancus carota* leaf meal (fDCLM), 2% fDCLM, 4% fDCLM, and 6% fDCLM.	The results indicated that inclusion of 2–6% fDCLM in the layer diet significantly increased laying rate egg, egg weight, and improved FCR compared to control. In addition, yolk colour, weight, and concentration of β-carotene in the yolk increased significantly in the fDCLM treatment group, while the yolk cholesterol concentration decreased than control.
Kothari et al. (2021)	40-week-old Hy-line brown laying hens	6 weeks	Dietary treatment groups: (1) basal diet + 0 mL fermented pine needle extract (FPNE)/kg diet (CON), (2) basal diet + 2.5 mL FPNE/kg diet (T1), or (3) basal diet + 5 mL FPNE/kg diet (T2).	Administration of fermented pine needle extract (FPNE) to 40-week-old laying hens for 42 days statistically increased laying rate and egg mass, feed intake and reduced Haugh unit. Notably, FPNE improved antioxidant activity of egg yolk after 6 weeks of storage. However, administration of FPNE at 2.5 and 5.0 mL/kg feed to layers had no effect on serum lipid profiles in layers.
Tian et al. (2022)	34-week-old Xianju layers	8 weeks	Dietary treatments: T1 = basal diet without lactobacillus fermented herbs (LFH) (CON group) and T2 = CON diet supplemented with 500 mg/kg of LFH. .	Laying hens fed basal diet supplemented with 500 mg/kg of lactobacillus fermented herbs (LFH) had increased laying rate, egg mass, albumen height, and Haugh unit from 38–42 weeks of age compared to layers fed basal diet without LFH supplementation. In addition, layers fed with the diet containing 500 mg/kg LFH had significantly higher albumen height and Haugh unit than those fed the control diet. Similarly, LFH supplementation significantly increased the villus height (VH) and crypt depth (CD) in the jejunum of laying hens, as well as the ratio of VH to CD.
Niu et al. (2023)	Hy-Line Brown hens (age, 23 weeks)	9 weeks	T1 = Basal diet without fermented *B. papyrifera* leaf meal (fBPLM) supplementation and T2 = Basal diet + 1% or 5% fBPLM supplementation.	The results suggested that fBPLM supplementation at 1.0% increased feed intake, FCR, and egg weight in layers. Similarly, dietary supplementation of fBPLM enhanced the egg yolk colour, but decreased the eggshell weight, eggshell thickness, and serum triglycerides. In contrast, fBPLM increased the content of high-density lipoprotein-cholesterol

Niu et al. (2023) found increased feed intake, egg weight, and poor FCR in layers fed 1 and 5% *Broussonetia papyrifera* fermented with *Lactobacillus plantarum*. The increased egg weights in layers offered TLMs were consistent with the results of other investigators (Abou-Elkhair et al. 2018; Lee et al. 2019). The mechanism behind the increased egg weight in layers fed fermented *B. papyrifera* supplementation is not clear but may be ascribed to the quality of the diet as fermented plant products are rich in nutrients and bioactive compounds (Shi et al. 2021; Sugiharto 2021).

Zhao et al. (2013) found that the total SFA level in egg yolk declined in response to fGLM supplementation. This decrease was principally due to the reduction in palmitic acid (C16:0) and stearic acid (C18:0). In the same study, the authors found that total PUFA was enhanced in response to dietary fGLM supplementation, which was primarily due to an increase in linoleic acid (C18:2) and arachidonic acid (C20:4) levels. A positive correlation exists between intake of SFA rich food and cardiovascular diseases, so a decrease in SFA in eggs from layers fed fermented TLMs appears beneficial (Salma et al. 2011).

### Challenges and opportunities of fermented tropical leaf meals in commercial poultry production

Information on the nutrient content and ANFs of fermented TLMs has been documented in the literature (Mat et al. 2020; Shi et al. 2021; Sugiharto et al. 2020). Variations in nutrient levels in fermented TLMs have also been established (Shi et al. 2021; Sugiharto et al. 2020). This difference is caused by a variety of factors, including fermentation conditions, fermentation type, nature, microbes used, and properties of the substrates used (Aladi et al. 2017; Sugiharto and Ranjitkar 2019). As a result, fermentation conditions, fermentation type, nature, microbes used, and properties of the substrates need to be established in order to optimise fermentation outcomes for target qualities for each tropical leaf meal. Various values for phytochemicals and macronutrients have been reported by various authors, as shown in Table 3, emphasising the need for standardisation of the nutrient content of various TLMs as to enhance the chance of their adoption in the poultry industry. Fermented tropical leaves are rich in fibre, but low in crude protein and ether extract values (Table 2) when compared to legumes like groundnut and soybean (Sarkiyayi and Agar 2010). Fermented tropical leaves have lower energy content than maize. Despite the potential for fermentation to improve the nutrient content and functional properties of TLMs (Shi et al. 2021; Sugiharto et al. 2020), the use of fermented TLMs has not been fully adopted by the commercial poultry feed industry due to inconsistencies in chicken responses when included in diets. This heterogeneity might be attributed to variables such as diet composition, type of leaf meal used, inclusion level, and environmental conditions. While there are no consistent agreements on the feeding quality of fermented TLMs in chicken feeding, fermentation holds promise for increased utilisation of TLMs in chicken nutrition.

## Conclusion

This review found that tropical leaves are rich in nutrients and important phytochemical compounds, but the use of their leaf meal in chicken nutrition is limited by their high fibre content and the presence of anti-nutritional components. On the other hand, the current research found that fermentation enhanced the nutrient and phytochemical composition of tropical leaf meals while decreasing their fibre and antinutrient content. This review found improved performance in broilers and laying hens fed moderate amounts of fermented tropical leaf meals. Despite numerous studies on the potential of fermentation to increase the nutrient content of tropical leaf meals, there is still a scanty literature on its safety and shelf-life. Taking this into account, it is recommended that further research be directed in this area. Further study should be conducted to establish the economics of tropical leaf meals when compared to conventional ingredients on an industrial scale to enhance their use in the poultry industry.

## Data Availability

Data will be made available on reasonable request.

## References

[CR1] Abduh MY, Yulianto R, Avima D, Widianto AK, Alfianny R (2021) Solid-state fermentation of cinnamon bark using *aspergillus awamori* to increase cinnamon oil yield extracted using hydrodistillation, maceration, and soxhlet extraction. Nat Volat Essent Oils 8:1575–1588

[CR2] Abou-Elkhair R, Selim S, Hussein E (2018) Effect of supplementing layer hen diet with phytogenic feed additives on laying performance, egg quality, egg lipid peroxidation and blood biochemical constituents. Anim Nut 4:394–400 10.1016/j.aninu.2018.05.009 PMC6286622 30564759

[CR3] Achonwa CC, Ogbuewu IP, Uchegbu MC, Okoli IC (2019) Physicochemical characteristics of *Ficus microcarpa* leaf meals harvested in southeastern Nigeria. Asian J Biol Scs 12:682–692

[CR4] Ahiwe EU, Ejiofor I, Oladipupo OA, Ogbuewu IP, Aladi NO, Obikaonu HO, Emenalom OO (2022) Effect of composite enzyme supplementation on production parameters, intestinal segment measurements, and apparent nutrient digestibility of broiler chickens fed low energy and protein diets. Trop Anim Hlth Prod 54:399 10.1007/s11250-022-03402-5 36422722

[CR5] Aladi NO, Chukwukaelo AA, Okeudo NJ, Ogbuewu IP, Ugwu CC, Etuk EB, Okoli IC (2017) Blood chemistry, haematology and ileal bacteria counts of broilers fed fermented mixtures of cassava root meal and palm kernel cake. Comp Clin Path 26:1273–1278

[CR7] Ali HSH, Fry JR, Abu BMF (2013) Phytochemicals content, antioxidant activity and acetylcholinesterase inhibition properties of indigenous *Garcinia parvifolia* fruit. BioMed Res Int 2013:138950 10.1155/2013/138950 PMC3832992 24288662

[CR8] Ali M, Kenganora M, Manjula SN (2016) Health benefits of *Morinda citrifolia* (Noni): a review. Pharmacog J 8:321–334

[CR6] Ali BA, Alfa AA, Tijani KB, Idris ET, Unoyiza US, Junaidu Y (2020) Nutritional health benefits and bioactive compounds of *Mangifera indica* L (mango) leaves methanolic extracts. Asian Plant Res J 6:41–51

[CR9] Antara KJ, Bidura GNG, Siti NW (2019) Effects of *Moringa oleifera* leaf and probiotics mixed fermented extract on the egg production and cholesterol contents in egg of laying hens. Int J Fauna Biol Stud 6:06–12

[CR10] Bao W, Huang X, Liu J, Han B, Chen J (2020) Influence of *Lactobacillus brevis* on metabolite changes in bacteria-fermented sufu. J Food Sci 85:165–172 31898817 10.1111/1750-3841.14968

[CR11] Belewu MA, Sam R (2010) Solid state fermentation of *Jatropha curcas* kernel cake: proximate composition and anti-nutritional components. J Yeast Fungal Res 1:44–46

[CR12] Bhanja T, Dey TB, Chakraborty S, Jain KK, Sharma A, Kuhad RC (2016) Antioxidant phenolics and their microbial production by submerged and solid-state fermentation process: a review. Trends Food Sci Techn 53:60–74

[CR13] Bidura GNG, Siti NW, Wibawa AAPP, Ariana NT, Puspani E (2021) The effect of carrot leaves meal fermented in diets on egg production, yolk cholesterol and beta-carotene in yolk of hens. Annals Rom Soc Cell Biol 25:18705–18711

[CR15] Canibe N, Jense BB (2021) Fermented liquid feed-microbial microbial and nutritional aspects and impact on enteric diseases in pigs. Anim Feed Sci Techn 173:17–40

[CR14] Canibe N, Højberg O, Badsberg JH, Jensen BB (2007) Effect of feeding fermented liquid feed and fermented grain on gastrointestinal ecology and growth performance in piglets. J Anim Sci 85:2959–2971 17591711 10.2527/jas.2006-744

[CR16] Cao FL, Zhang XH, Yu WW, Zhao LG, Wang T (2012) Effect of feeding fermented *Ginkgo biloba* leaves on growth performance, meat quality, and lipid metabolism in broilers. Poult Sci 91:1210–1221 22499881 10.3382/ps.2011-01886

[CR17] Cayan H, Erener G (2015) Effect of olive leaf (*Olea europaea*) powder on laying hens’ performance, egg quality and egg yolk cholesterol levels. Asian Austral J Anim Sci 28:538–543 10.5713/ajas.14.0369 PMC4341103 25656181

[CR18] Chukwukaelo AK, Aladi NO, Okeudo NJ, Obikaonu HO, Ogbuewu IP, Okoli IC (2018) Performance and meat quality characteristics of broilers fed fermented mixtures of grated cassava roots and palm kernel cake as replacement for maize. Trop Anim Hlth Prod 50:485–493 10.1007/s11250-017-1457-7 29098536

[CR19] Dey TB, Chakraborty S, Jain KK, Sharma A (2016) Antioxidant phenolics and their microbial production by submerged and solid-state fermentation process: a review. Trends Food Sci Techn 53:60–74

[CR20] Ding Y, Jiang X, Yao X, Zhang H, Song Z, He X, Cao R (2021) Effects of feeding fermented mulberry leaf powder on growth performance, slaughter performance, and meat quality in chicken broilers. Animals 11:3294 34828025 10.3390/ani11113294 PMC8614317

[CR21] Du Y (2020) Endocrine and genetic factors affecting egg laying performance in chickens: a review. Brit Poult Sci 61:538–549 32306752 10.1080/00071668.2020.1758299

[CR22] Dulf FV, Vodnar DC, Socaciu C (2016) Effects of solid-state fermentation with two filamentous fungi on the total phenolic contents, flavonoids, antioxidant activities and lipid fractions of plum fruit (*Prunus domestica* L.) by-products. Food Chem 209:27–36 27173530 10.1016/j.foodchem.2016.04.016

[CR23] Engberg RM, Hammershøj M, Johansen NF, Abousekken MS, Steenfeldt S, Jensen BB (2009) Fermented feed for laying hens: effects on egg production, egg quality, plumage condition and composition and activity of the intestinal microflora. Brit Poult Sci 50:228–239 19373724 10.1080/00071660902736722

[CR25] Esonu BO, Iheukwumere FC, Iwuji TC, Akanu N, Nwugo OH (2003) Evaluation of *Microdesmis puberula* leaf meal as feed ingredients in broiler starter diets. Nig J Anim Prod 30:3–8

[CR24] Esonu BO, Azubuike JC, Emenalom OO, Etuk EB, Okoli IC, Ukwu H, Nneji CS (2004) Effect of enzyme supplementation on the performance of broiler finisher fed Microdesmis puberula leaf meal. Int J Poult Sci 3:112–114

[CR26] Etuk EB, Ugwu CC, Inyama E, Ugorji C (2014) Blood chemistry, haematology, carcass characteristics and organ weight of finisher broilers fed breadfruit (*Treculia africana*) hull (BFH) in their diet. Comp Clin Path 23:1153–1158

[CR27] Falowo AB, Mukumbo FE, Idamokoro EM, Lorenzo JM, Afolayan AJ, Muchenje V (2018) Multi-functional application of *Moringa oleifera* Lam. In nutrition and animal food products: a review. Food Res Int 106:317–334 29579932 10.1016/j.foodres.2017.12.079

[CR28] Grashorn MA (2010) Use of phytobiotics in broiler nutrition: an alternative to infeed antibiotics. J Anim Feed Sci 19:338–347

[CR29] Hassaan MS, Soltan MA, Abdel-Moez AM (2015) Nutritive value of soybean meal after solid state fermentation with *Saccharomyces cerevisiae* for Nile tilapia, *Oreochromis niloticus*. Anim Feed Sci Techn 201:89–98

[CR30] Ifesan BOT, Egbewole OO, Ifesan BT (2014) Effect of fermentation on nutritional composition of selected commonly consumed green leafy vegetables in Nigeria. Int J Appld Sci Biotechn 2:291–297

[CR31] Ijarotimi OS, Adeoti OA, Ariyo O (2013) Comparative study on nutrient composition, phytochemical, and functional characteristics of raw, germinated, and fermented *Moringa oleifera* seed flour. Food Sci Nut 1:452–463 10.1002/fsn3.70 PMC3951542 24804056

[CR32] Jha R, Mishra P (2021) Dietary fibre in poultry nutrition and their effects on nutrient utilization, performance, gut health, and on the environment: a review. J Anim Sci Biotechn 12:1–16 10.1186/s40104-021-00576-0 PMC8054369 33866972

[CR33] Kiczorowski P, Kiczorowska B, Samolińska W, Szmigielski M, WiniarskaMieczan A (2022) Effect of fermentation of chosen vegetables on the nutrient, mineral, and biocomponent profile in human and animal nutrition. Sci Rep 12:13422 35927577 10.1038/s41598-022-17782-z PMC9352655

[CR34] Kim HJ, Oh GT, Park YB, Lee MK, Seo HJ, Choi MS (2004) Naringin alters the cholesterol biosynthesis and antioxidant enzyme activities in LDL receptor-knockout mice under cholesterol fed condition. Life Sci 74:1621–1634 14738906 10.1016/j.lfs.2003.08.026

[CR35] Kim YJ, Rubayet Bostami ABM, Islam MM, Mun HS, Ko SY, Yang CJ (2017) Performance, immunity, meat composition and fatty acid pattern in broilers after dietary supplementation of fermented *Ginkgo biloba* and *Citrus junos*. J Nut Food Sci 7:591

[CR36] Knez E, Kadac-Czapska K, Grembecka M (2023) Effect of fermentation on the nutritional quality of the selected vegetables and legumes and their health effects. Life 13:655 36983811 10.3390/life13030655 PMC10051273

[CR37] Kothari D, Oh JS, Kim JM, Lee WD, Kim SK (2021) Effect of dietary supplementation of fermented pine needle extract on productive performance, egg quality, and serum lipid parameters in laying hens. Anim (Basel) 11:1475 10.3390/ani11051475 PMC8161186 34065559

[CR38] Lee AR, Niu KM, Lee WD, Kothari D, Kim SK (2019) Comparison of the dietary supplementation of Lactobacillus plantarum, and fermented and non-fermented *Artemisia annua* on the performance, egg quality, serum cholesterol, and egg yolk-oxidative stability during storage in laying hens. Braz J Poult Sci 21:001–008

[CR39] Liu AH, Bao YM, Wang XY, Zhang ZX (2013) Cardioprotection by *Ginkgo biloba* extract 50 in rats with acute myocardial infarction is related to Na^+^-Ca^2+^ exchanger. Amer J Chin Med 41:789–800 23895152 10.1142/S0192415X13500535

[CR40] Liu L, Wang Y, Zhang J, Wang S (2021) Advances in the chemical constituents and chemical analysis of *Ginkgo biloba* leaf, extract, and phytopharmaceuticals. J Pharmaceut Biomed Analy 193:113704 10.1016/j.jpba.2020.113704 33157480

[CR41] Londero A, Pelaez MAL, Diosma G, De Antoni GL, Abraham AG, Garrote GL (2014) Fermented whey as poultry feed additive to prevent fungal contamination. J Sci Food Agric 94:3189–3194 24652751 10.1002/jsfa.6669

[CR42] Mandey JS, Leke JR, Kaunang WB, Kowel YHS (2015) Carcass yield of broiler chickens fed banana (*Musa Paradisiaca*) leaves fermented with *Trichoderma Viride*. J Indo Trop Anim Agric 40:229–233

[CR43] Manihuruk FH, Ismail I, Rastina R, Razali R, Sabri M, Zuhrawati Z, Muhammad M, Jalaluddin J (2018) Effect of fermented Moringa (*Moringa oleifera*) leaf powder in feed to increase broiler carcass weight. J Medika Vet 12:103–109

[CR44] Mapelli-Brahm P, Barba FJ, Remize F, Garcia C, Fessard A, Khaneghah AM, Sant’ana AS, Lorenzo JM, Montesano D, Meléndez-Martínez AJ (2020) The impact of fermentation processes on the production, retention and bioavailability of carotenoids: an overview. Trends Food Sci Techn 99:389–401

[CR45] Mat K, Taufik HA, Rusli ND, Hasnita CH, Al-Amsyar SM, Rahman MM, Mohd M (2020) Effects of fermentation on the nutritional composition, mineral content and physical characteristics of banana leaves. IOP Conf. Series: Earth and Envntal Sci 596: 012089

[CR46] Melini F, Melini V, Luziatelli F, Ficca AG, Ruzzi M (2019) Health-promoting components in fermented foods: an up-to-date systematic review. Nutrients 11:1189 31137859 10.3390/nu11051189 PMC6567126

[CR47] Missotten JA, Michiels J, Dierick Nl, Ovyn A, Akbarian A, De Smet S (2013) Effect of fermented moist feed on performance, gut bacteria and gut histomorphology in broilers. Brit Poult Sci 54:627–634 23927009 10.1080/00071668.2013.811718

[CR48] Modisaojang-Mojanaja MMC, Ogbuewu IP, Mokolopi BG, Oguttu JW, Mbajiorgu CA (2019) Mineral composition of *Moringa oleifera* leaf meal (MOLM) and the response of Ross 308 broilers to MOLM supplementation. Appld Ecol Environ Res 17:8139–8150

[CR49] Ndubuaku UM, Nwankwo VU, Baiyeri KP (2014) Influence of poultry manure application on the leaf amino acid profile, growth and yield of moringa (*Moringa oleifera* Lam.) Plants. Albanian J Agric Sci 13:42

[CR52] Niu Y, Wan XL, Zhang XH, Zhao LG, He JT, Zhang JF, Zhang LL, Wang T (2017) Effect of supplemental fermented *Ginkgo biloba* leaves at different levels on growth performance, meat quality, and antioxidant status of breast and thigh muscles in broiler chickens. Poult Sci 96:869–877 27664198 10.3382/ps/pew313

[CR53] Niu Y, Zhang JF, Wan XL, Huang Q, He JT, Zhang XH, Zhao LG, Zhang LL, Wang T (2019) Effect of fermented *Ginkgo biloba* leaves on nutrient utilisation, intestinal digestive function and antioxidant capacity in broilers. Brit Poult Sci 60:47–55 30345798 10.1080/00071668.2018.1535166

[CR51] Niu KM, Khosravic S, Wang Y, Zhai Z, Wang R, Liu J, Cai L, Li J, Deng L, Wu X (2022) Multiomics-based functional characterization of hybrid fermented *Broussonetia papyrifera*: a preliminary study on gut health of laying hens. Fermentation 8:547

[CR50] Niu K, Wang YF, Liang X, Zhai Z, Liu J, Wang R, Chen G, Wu X (2023) Impact of fermented *Broussonetia papyrifera* on laying performance, egg quality, lipid metabolism, and follicular development of laying hens. Poult Sci 102:102569 36913757 10.1016/j.psj.2023.102569 PMC10023956

[CR54] Nordrum S, Bakke-McKellep A, Krogdahl Å, Buddington R (2000) Effects of soybean meal and salinity on intestinal transport of nutrients in Atlantic salmon (*Salmo salar* L.) and rainbow trout (*Oncorhynchus mykiss*). Comp Biochem Physiol 125:317–335 10.1016/s0305-0491(99)00190-x 10818266

[CR55] Nwosu OK, Ubaoji KI, Okaka ANC (2018) Evaluation of nutritional and anti-nutritional compositions of leaves of (Maiden Hair) tree found in Nigeria *Gingko biloba*. J Exptal Res 6:66–72

[CR60] Ogbuewu IP, Mbajiorgu CA (2019) Potential of leaf and seed meals of tropical plants in chicken diet: effect on spermatozoa and egg production. Trop Anim Hlth Prod 51:267–277 10.1007/s11250-018-1715-3 30251210

[CR57] Ogbuewu IP, Mbajiorgu CA (2023a) Review of selected trace elements in tropical medicinal plants used as feedstuffs and additives in animal production. Appld Ecol Envntal Res 21:3791–3805

[CR58] Ogbuewu IP, Mbajiorgu CA (2023b) Meta-analysis of health status and production physiology of broiler chickens on dietary fermented cassava intervention. Trop Anim Hlth Prod 55:368 10.1007/s11250-023-03783-1 PMC10590304 37864719

[CR59] Ogbuewu IP, Mbajiorgu CA (2023c) Dietary *Dialium guineense* stem-bark supplementation improves growth performance and haemato-biochemical characteristics of broiler chickens. Heliyon 9:e17341 37484235 10.1016/j.heliyon.2023.e17341 PMC10361381

[CR62] Ogbuewu IP, Mbajiorgu CA (2023d) Dose-related responses of broiler chickens to black velvet tamarind (*Dialium guineense*) stem bark supplementation: carcass characteristics, organ weight and intestinal biometry. Agroforest Syst 98:245–254. 10.1007/s10457-023-00902-7

[CR56] Ogbuewu IP, Emenalom OO, Okoli IC (2015) Alternative feedstuffs and their effects on blood chemistry and haematology of rabbits and chickens: a review. Comp Clin Path 26:277–286

[CR61] Ogbuewu IP, Modisaojang-Mojanaga MMC, Mokolopi BG, Mbajiorgu CA (2023) Nutritional and chemical composition of Black velvet tamarind (*Dialium guineense* Wild) and its influence in animal production: a review. Open Agric 8:20220174

[CR63] Ojokoh AO (2007) Effect of fermentation on the chemical composition of mango (*Mangifera indica* R) peels. Afr J Biotechn 6:1979–1981

[CR64] Okeke UB, Adeboye OM, Adeniyi FR, Agbebi EA (2023) A review on ethnobotany, phytochemistry, and pharmacology of *Microdesmis Keayana* and *Microdesmis puberula* (Pandaceae). J Appld Pharmaceut Sci 13:001–013

[CR66] Okoli C, Okoli IC, Emenalom OO, Esonu BO, Udedibie ABI (2014) The emerging nutraceutical benefits of the African wonder nut (*Garcinia kola* Heckel): a review. Global J Anim Sci Res 2:170–183

[CR65] Okoli IC, Udedibie COI, Achonwa CC, Ogbuewu IP, Anyanwu NJ, Enemor VHA (2019) Physicochemical characterizations of leaf meals derived from tropical plants as possible nutraceuticals in animal production. Asian J Biol Sci 12:693–701

[CR67] Park J, Song T, Kim I (2016) Egg production, egg quality, and cecal microbial populations of layers fed diets supplemented with fermented phytogenic feed additive. Turkish J Vet Anim Sci 40:660–666

[CR68] Prasad MNV, Freitas H (2000) Removal of toxic metals from solution by leaf, stem and root phytomass of *Quercus ilex* L. (holly oak). Envntal Pollution 110:277–283 10.1016/s0269-7491(99)00306-1 15092842

[CR69] Princewill-Ogbonna IL, Ogbonna PC, Ogujiofor IB (2019) Proximate composition, vitamin, mineral and biologically active compounds levels in leaves of *Mangifera indica* (Mango), *Persea americana* (Avocado pea), and *Annona muricata* (soursop). J Appld Sci Envntal Mgt 23:65–74

[CR70] Puvaca N, Tufarelli V, Giannenas I (2022) Essential oils in broiler chicken production, immunity and meat quality: review of *Thymus vulgaris*, *Origanum vulgare* and *Rosmarinus officinalis*. Agric 12:874

[CR71] Ranjitkar S, Engberg RG (2016) The influence of feeding crimped kernel maize silage on growth performance and intestinal colonization with *Campylobacter jejuni* in broilers. Avian Path 45:253–560 27100153 10.1080/03079457.2016.1146821

[CR72] Rasaq I (2017) Utilization of fermented *Mucuna pruriens* leaf meal as a replacement for soyabean meal in *Clarias gariepinus* (Burchell, 1822) fingerlings diets. MSc Thesis, Ahmadu Bello University, Zaria. Pp. 1–91

[CR73] Sachindra NM, Bhaskar N (2008) In vitro antioxidant activity of liquor from fermented shrimp biowaste. Biores Techn 99:9013–9016 10.1016/j.biortech.2008.04.036 18513957

[CR74] Salma U, Miah AG, Tsujii H, Schellander K, SuDekum KH (2011) Effect of dietary *Rhodobacter capsulatus* on lipid fractions and egg-yolk fatty acid composition in laying hens. J Anim Physiol Anim Nut (Berl) 96:1091–1100 10.1111/j.1439-0396.2011.01224.x 21929698

[CR75] Santoso U, Fenita Y, Biudra IGNG (2015a) Effect of fermented *Sauropus androgynus* leaves on meat composition, amino acid and fatty acid compositions in broiler chickens. Pak J Nut 14:799–807

[CR76] Santoso U, Fenita Y, Kususiyah K (2015b) Effect of fermented *Sauropus androgynus* leaves on blood lipid fraction and haematological profile in broiler chickens. J Indonesian Trop Anim Agric 40:199–207

[CR77] Santoso U, Fenita Y, Kususiyah K (2018) The Effect of fermented *Sauropus androgynus* plus bay leaf inclusion on the hematologic and lipid profiles of female broiler chickens. Int J Poult Sci 17:410–417

[CR78] Sarkiyayi S, Agar TM (2010) Comparative analysis on the nutritional and anti-nutritional contents of the sweet and bitter cassava varieties. Adv J Food Sci Techn 2:328–334

[CR79] Sati SC, Joshi S (2011) Antibacterial activities of *Ginkgo biloba* L. leaf extracts. Sci World J 11:2237–2242 10.1100/2011/545421 PMC3221511 22125471

[CR80] Sebola NA, Mlambo V, Mokoboki HK, Muchenje V (2015) Growth performance and carcass characteristics of three chicken strains in response to incremental levels of dietary *Moringa oleifera* leaf meal. Livest Sci 178:202–208

[CR82] Shi H, Yang E, Li Y, Chen X, Zhang J (2021) Effect of solid-state fermentation on nutritional quality of leaf flour of the drumstick tree (*Moringa oleifera* Lam). Front Bioeng Biotechn 9:626628 10.3389/fbioe.2021.626628 PMC8072291 33912544

[CR81] Shih MC, Chang CM, Kang SM, Tsai ML (2011) Effect of different parts (leaf, stem and stalk) and seasons (summer and winter) on the chemical compositions and antioxidant activity of *Moringa oleifera*. Int J Mol Sci 12:6077–6088 22016645 10.3390/ijms12096077 PMC3189769

[CR83] Shim YH, Shinde PL, Choi JY, Kim JS, Seo DK, Pak JI, Chae BJ, Kwon IK (2010) Evaluation of multimicrobial probiotics produced by submerged liquid and solid substrate fermentation methods in broilers. Asian Austral J Anim Sci 23:521–529

[CR84] Siaboc NV (2018) Performance attributes of broiler chicken (*Gallus gallus Domesticus*) supplemented with fermented jute leaves (*Corchorus olitorius*) under camiguin condition. J Multidiscipl Stud 7:85–102

[CR85] Singh B, Kaur P, Singh GRD, Ahuja PS (2008) Biology and chemistry of *Ginkgo biloba*. Fitoterapia 79:401–418 18639617 10.1016/j.fitote.2008.05.007

[CR86] Siti NW, Bidura IGNG (2018) Effect of fermented *Moringa oleifera* leaf extract in drinking water on egg production, yolk colour, serum and egg cholesterol levels in laying chicken. Book of Proceedings of International Conference on Science, Technology and Humanities held on October 22–23, 2018 at Kuta Bali. pp 1–10

[CR87] Siti N, Bidura IGNG, Putri UIA (2017) The Effect of water extract of leaves *Moringa oleifera* on egg production and yolk cholesterol levels in egg laying hens. J Biol Chem Res 34:657–665

[CR88] Sokrab AM, Ahmed IAM, Babiker EE (2014) Effect of fermentation on antinutrients, and total and extractable minerals of high and low phytate corn genotypes. J Food Sci Techn 51:2608–2615 10.1007/s13197-012-0787-8 PMC4190255 25328202

[CR89] Sugiharto S (2021) Fermented leaves in broiler rations: effects on growth performance, physiological condition, and meat characteristics. Acta Vet Eurasia 47:44–50

[CR90] Sugiharto S, Ranjitkar S (2019) Recent advances in fermented feeds towards improved broiler chicken performance, gastrointestinal tract microecology and immune responses: a review. Anim Nut 5:1–10 10.1016/j.aninu.2018.11.001 PMC6407077 30899804

[CR91] Sugiharto S, Lauridsen C, Jensen BB (2015) Gastrointestinal ecosystem and immunological responses in E. Coli challenged pigs after weaning fed liquid diets containing whey permeate fermented with different lactic acid bacteria. Anim Feed Sci Techn 207:278–282

[CR92] Sugiharto S, Widiastuti E, Isroli I, Yudiarti T, Sartono TA, Wahyuni HI (2020) Breast meat characteristics of broilers fed fermented mixture of cassava pulp and Moringa oleifera leaf meal. J Indonesian Trop Anim Agric 45:103–114

[CR93] Supriyati HT, Susanti T, Susana IWR (2015) Nutritional value of rice bran fermented by *Bacillus amyloliquefaciens* and humic substances and its utilization as a feed ingredient for broiler chickens. Asian Austral J Anim Sci 28:231–238 10.5713/ajas.14.0039 PMC4283168 25557819

[CR94] Syahruddin E, Abbas H, Purwati E, Heryandi Y (2011) Effect of fermented noni leaf (*Morinda citrifolia* L.) in diets on cholesterol content of broiler chicken carcass. Jurnal Ilmu Ternak Dan Veteriner 16:266–271

[CR96] Syahruddin E, Herawaty R, Ningrat RWS (2013) Effect of fermented katuk leaf (*Sauropus androgynus* L. Merr.) In diets on cholesterol content of broiler chicken carcass. Pak J Nut 12:1013–1018

[CR95] Syahruddin E, Herawaty R, Ahzar RW (2016) Effect of substituting soybean meal with fermented rubber leaves and seeds (*Hevea brasilliensis*) on egg production and egg quality in native laying hens. Int J Poult Sci 15:325–329

[CR97] Tefera T, Ameha K, Biruhtesfa A (2014) Cassava based foods: microbial fermentation by single starter culture towards cyanide reduction, protein enhancement and palatability. Int Food Res J 21:1751–1756

[CR98] Tian Y, Li G, Zhang S, Zeng T, Chen L, Tao Z, Lu L (2022) Dietary supplementation with fermented plant product modulates production performance, egg quality, intestinal mucosal barrier, and cecal microbiota in laying hens. Front Microbiol 13:955115 36246237 10.3389/fmicb.2022.955115 PMC9561940

[CR99] Ting S, Yeh HS, Lien TF (2011) Effects of supplemental levels of hesperetin and naringenin on egg quality, serum traits and antioxidant activity of laying hens. Anim Feed Sci Techn 163:59–66

[CR100] Udo UE, Udo AU, Dan EU (2017) Determination of nutrient, antinutrient compositions and median lethal dose of leaves of *Microdesmis puberula* grown in Nigeria. J Sci Res Rep 17:1–10

[CR101] Umaru HA, Adamu R, Dahiru D, Nadro MS (2007) Levels of antinutritional factors in some wild edible fruits of Northern Nigeria. Afr J Biotechn 6:1935–1938

[CR102] van Beek TA, Montoro P (2009) Chemical analysis and quality control of *Ginkgo biloba* leaves, extracts, and phytopharmaceuticals. J Chromato A 1216:2002–2032 10.1016/j.chroma.2009.01.013 19195661

[CR103] Wang J, Cao F, Zhu Z, Zhang X, Sheng Q, Qin W (2018) Improvement of quality and digestibility of *Moringa oleifera* leaves feed via solid-state fermentation by Aspergillus Niger. Int J Chem Reactor Eng 16:12

[CR104] Wang Y, Wu J, Lv M, Shao Z, Hungwe M, Wang J, Bai X, Xie J, Wang Y, Geng W (2021) Metabolism characteristics of lactic acid bacteria and the expanding applications in food industry. Frontier Bioeng Biotechnol 9:612285 10.3389/fbioe.2021.612285 PMC8149962 34055755

[CR105] Woke GN, Aleleye-Wokoma IP, Komi GW, Bekibele DO (2013) Effect of fermented and unfermented Mucuna bean seed on growth performance of Tilapia. Glob J Pure Appld Scs19:9–15

[CR106] Xie PJ, Huang LX, Zhang CH, Zhang YL (2016) Nutrient assessment of olive leaf residues processed by solid-state fermentation as an innovative feedstuff additive. J Appld Microbiol 121:28–40 10.1111/jam.13131 26991541

[CR107] Xie M, Wang R, Wang Y, Liu N, Qi J (2021) Effects of dietary supplementation with fermented *Chenopodium album* L. on growth, nutrient digestibility, immunity, carcass characteristics and meat quality of broilers. Italian J Anim Sci 20:2063–2074

[CR108] Yamamoto T, Iwashita Y, Matsunari H, Sugita T, Furuita H, Akimoto A, Okamatsu K, Suzuki N (2010) Influence of fermentation conditions for soybean meal in a non-fish meal diet on the growth performance and physiological condition of rainbow trout (*Oncorhynchus mykiss*). Aquacu 309:173–180

[CR109] Yang LJ, Yang ZB, Yang WR, Li HR, Zhang CY, Jiang SZ, Li XM (2018) Conventional solid fermentation alters mycotoxin contents and microbial diversity analyzed by high-throughput sequencing of a Fusarium mycotoxin-contaminated diet. Can J Anim Sci 98:354–361

[CR111] Yu W, Zhang X, Ahmad H, Zhao L, Wang T, Cao F (2015) Intestinal absorption function of broiler chicks supplemented with Ginkgo leaves fermented with *Bacillus species*. Pak J Zool 47:479–490

[CR110] Yu X, Su Q, Geng J (2021) *Ginkgo biloba* leaf extract prevents diabetic nephropathy through the suppression of tissue transglutaminase. Exptal Therapeutic Med 21:333 10.3892/etm.2021.9764 PMC7903480 33732306

[CR113] Zhang XH, Cao FL, Sun ZY, Yu WW, Zhao LG, Wang GB, Wang T (2012) Effect of feeding *Aspergillus Niger*-fermented *Ginkgo biloba*-leaves on growth, small intestinal structure and function of broiler chicks. Livest Sci 147:170–180

[CR114] Zhang XH, Sun ZY, Cao FL, Ahmad H, Yang XH, Zhao LG, Wang T (2015) Effects of dietary supplementation with fermented ginkgo leaves on antioxidant capacity, intestinal morphology and microbial ecology in broiler chicks. Brit Poult Sci 56:370–380 25868615 10.1080/00071668.2015.1030590

[CR112] Zhang X, Sun Z, Cai J, Wang G, Zhu Z, Zhao L, Cao F (2020) Dietary supplementation with fermented Radix astragalus-ginkgo leaves improves antioxidant capacity and meat quality in broilers. Pak J Zool 52:1571–1585

[CR115] Zhao LG, Zhang XH, Cao FL, Sun DF, Wang T, Wang GB (2013) Effect of dietary supplementation with fermented Ginkgo-leaves on performance, egg quality, lipid metabolism and egg-yolk fatty acids composition in laying hens. Livest Sci 155:77–85

[CR116] Zhou H, Wang C, Ye J, Chen H, Tao R (2015) Effects of dietary supplementation of fermented *Ginkgo biloba* L. residues on growth performance, nutrient digestibility, serum biochemical parameters and immune function in weaned piglets. Anim Sci J 86:790–799 25827443 10.1111/asj.12361

[CR117] Zulfan Z, Zulfikar Z, Latif H, Allaily A, Nazarullah T, Shaleha R (2021) Effects of using fermented moringa (Moringa oleifera) leaf meal and yellow corn in the diets on the performances and income over feed cost of the broiler chickens. J Agripet 21(1):84–91. 10.17969/agripet.v21i1.19804

